# Hip Joint Kinematic Covariation During Gait Before and 1-Year After Hip Arthroscopic Surgery for Femoroacetabular Impingement Syndrome

**DOI:** 10.3389/fsurg.2021.614329

**Published:** 2021-08-17

**Authors:** Philip Malloy, Donald Dr. Neumann, Anne Leung, Kristof Kipp

**Affiliations:** ^1^Department of Physical Therapy, Arcadia University, Glenside, PA, United States; ^2^Department of Orthopaedic Surgery, Rush University Medical Center, Chicago, IL, United States; ^3^Program in Exercise Science, Department of Physical Therapy, Marquette University, Milwaukee, WI, United States

**Keywords:** femoroacetabular impingement syndrome, biomechanics, gait analysis, hip, kinematic covariation

## Abstract

The primary aim of this study was to determine if the three-dimensional (3D) hip joint motion coordination during gait changes after arthroscopic surgery for femoroacetabular impingement syndrome (FAIS). Three-dimensional hip joint kinematic data were collected with a 12-camera motion capture system. Five trials of level walking were collected preoperatively (PRE) and at 1-year postoperatively (POST) in 8 patients diagnosed with FAIS and at a single time point in 8 healthy controls. Planar covariation analysis was performed to quantify the 3D hip joint motion coordination strategy during gait. Independent sample's *t*-test were used to determine differences between the FAIS group at the preoperative time point (PRE) and healthy controls. Paired samples *t*-tests were used to determine differences between the PRE and POST time points within the FAIS group. The %VAF by PC 1 for the FAIS group at the PRE time point was significantly less than that of healthy controls (PRE: 77.2 ± 8.7% vs. Control: 96.1 ± 2.8%; *p* = 0.0001), and the % VAF of the second PC (PC2) was significantly greater [PRE: 22.8 (8.7)%; Control: 3.9 (2.8)%; *p* = 0.0001]. No differences in %VAF were found between the PRE and POST time points within the FAIS group for PC1 [PRE: 77.2 (8.7)% vs. POST: 79.3 (11.1)%; *p* = 0.472] or PC2 [PRE: 22.7 (8.7)%; POST: 20.7 (11.1)%; *p* = 0.472]. Significant differences in the plane specific contribution to the 3D motion coordination strategy were found between the FAIS patients at the PRE and POST time points for the sagittal plane [PRE: 5.6 (2.7) vs. POST: 0.91 (6.1); *p* = 0.012] and frontal plane [PRE: −10.4 (2.2) and −1.5 (6.3); *p* = 0.005]. Patients with FAIS demonstrated a more complex coordination strategy of 3D hip joint motion than controls and this strategy remains unchanged after hip arthroscopic surgery despite changes in the plane specific contribution to this strategy. These findings indicate that motor control impairments in FAIS patients do exist and seem to persist for at least 1 year after hip arthroscopic surgery.

## Introduction

Femoroacetabular impingement syndrome (FAIS) causes hip related groin pain and is associated with abnormal bone shape of the proximal femur and/or acetabulum (1). This abnormal bone shape can cause symptomatic contact between the femur and acetabulum during normal hip motion, and has been implicated in the development of hip osteoarthritis (OA) through altered mechanical joint loading (1). Hip arthroscopy has emerged as a primary treatment for FAIS, which has grown exponentially in recent years (2–5). The rationale behind hip arthroscopy for FAIS is to reshape the proximal femur and/or acetabulum by removing the abnormal bone to restore joint congruence and biomechanics at the hip joint during function tasks. Outcomes following surgery have been reported as very good to excellent, when measured using hip specific patient reported outcomes (PROs) (3–5). However, the evidence remains limited on how hip arthroscopy influences joint level biomechanical function in patient with FAIS (6).

All previous studies examining gait kinematics in people with FAIS and before and after surgery have used a traditional analysis approach by statistically comparing discrete biomechanical variables (i.e., maximum or minimum values) in each plane of motion (7–11). A draw back to this analysis approach is that it eliminates the majority of biomechanical data collected during the task. A recent systematic review found that across studies, the main hip joint kinematic differences between people with FAIS and healthy controls are lower peak hip extension angles, less total sagittal plane hip joint range of motion and less peak internal rotation during the stance phase of walking (6). Although these studies establish that movement patterns during walking are different between people with FAIS and healthy controls, evidence on hip biomechanics before and after arthroscopic surgery for FAIS remains limited (6–11). Rylander and colleagues reported that following arthroscopic hip surgery for FAIS patients demonstrated improved sagittal plane and internal hip joint range of motion during walking (7, 8). However, more recent studies examining gait kinematics before and after hip arthroscopic surgery have reported no differences in hip kinematics after this surgical procedure (10, 11). Significantly reducing hip joint kinematic data using traditional analyses may be the potential reason for the inconsistency among these biomechanical studies before and after surgery for FAIS. Therefore, analysis methods that eliminate the need for an a priori selection of variables are needed to help better understand differences in biomechanics and motor control in people with FAIS before and after surgery.

The kinematic planar covariation framework provides a way to investigate aspects of motor control during gait through the application of advanced analysis methods such as principal components analysis (PCA) of biomechanical waveform data (12–16). PCA is a multivariate statistical technique that provides an unbiased way to extract a few principle modes of variation from a large data set (17). The concept of kinematic covariation emerged to shed light on how the nervous system controls walking by reducing the redundant degrees of freedom during the task (13, 14). The framework combines the motor control paradigms surrounding: (1) spinal neural networks (i.e., central pattern generators or CPGs) that generate patterns that drive rhythmic activity to produce cyclic limb motion, and (2) walking mechanics that pertain to center of mass control to promote stability and energy conservation (13). The application of PCA to the covariance matrix of kinematic waveform data during walking gait has revealed that the angular trajectories of the time varying segments covary such that these angles lie close to a plane, which is defined as kinematic covariation (13, 15, 16). During walking gait, the first two principal component (PC) have been shown to explain the greatest variation in 3D angular waveforms (~99% total variance) (13, 15). The orientation of the plane of angular covariance reflects the phase relationship of the angles, therefore represents the timing of 3D coordination amongst the angles (15). Ivanenko et al. found that during walking, the first PC represented thigh and shank segment coordination, where is variance accounts for basic limb movements responsible for limb length and orientation (15). Conversely, specific end point control related to foot placement, which is thought to vary more so during gait, and was most related to variance explained by the second PC (15). As such, a kinematic covariation strategy where a single PC alone explains >90% of the total variance is referred to as linear strategy, whereas a kinematic covariation strategy where two PCs are needed to explain the majority or all of the variance in the kinematic waveforms is termed a planar strategy of kinematic covariation (15, 18).

The kinematic covariation framework has been applied to three-dimensional (3D) joint angles to assess kinematic control in people with chronic ankle sprains (19), however, not in the context of hip conditions. Very little information exists on the study of motor control paradigms related to FAIS, despite many of the non-operative treatments for this condition addressing movement control (20–22). The application of the kinematic covariation frame work to gait before and after surgery for FAIS provides insight into how this condition and treatment may impact motor control in these patients. The two aims of this exploratory study were: (1) to determine if kinematic covariation of the 3D hip joint angles during walking gait is different between people with FAIS and healthy controls, and (2) to determine if this strategy changes within FAIS patients after arthroscopic hip surgery. It is hypothesized that persons with FAIS will demonstrate a different kinematic covariation strategy at the hip joint compared to healthy controls, and that this strategy will change within FAIS patients after arthroscopic hip.

## Methods

### Study Design and Study Sample

The original study design from which the data for this study were collected was a large cross sectional study comparing hip biomechanical function in people with FAIS (*n* = 20) and healthy controls (*n* = 20) (23). This exploratory study was conducted on a convenience subsample of 8 FAIS patients who elected to undergo surgery from the larger cross-sectional cohort, and agreed to return for follow-up postoperative testing. As such an a priori power analysis was not conducted for this exploratory investigation. All subjects returned for follow-up testing between 12–18 months after surgery with the average (± standard deviation) length of time between the preoperative and postoperative time point being 15.2 ± 3.9 months. Eight participants with FAIS undergoing hip arthroscopy and 8 healthy age-and-sex matched controls were included in this study (Table 1). Participants with FAIS undergoing surgery included in this study were collected at two time points, the preoperative time point was <14 days prior to hip arthroscopic surgery, and the postoperative data was collected at the >12-months following hip arthroscopic surgery for FAIS time point. Data on control participants were collected at a single-time point. This study was approved by the local universities office of research compliance. All participants provided a written informed consent before participation in the study.

**Table 1:** Demographics for the FAIS group at the preoperative (PRE) and postoperative (POST) time points and controls.

	**PRE**	**POST**	**Control**	***P***	***P***
	(***n*** **= 8)**	(***n*** **= 8)**	(***n*** **= 8)**	**(Pre. vs. Control)**	**(Pre. vs. Post)**
Gender	2F, 6M	2F,6M	2F, 6M	–	–
Age (Y)	22.3 (6.9)	23.4 (6.8)	22.3 (3.5)	0.964	–
Height (m.)	1.76 (0.08)	1.78 (0.10)	1.77 (0.10)	0.694	0.123
Body Mass (kg.)	76.0 (10.8)	78.4 (12.1)	74.3 (12.1)	0.769	0.091

All FAIS participants were recruited from a private orthopedic surgery practice of a single surgeon who specializes in the treatment of hip disorders. All healthy age-and-sex matched controls were recruited from a general university population. FAIS diagnosis was based on the clinical signs of hip pain lasting >3 months, a positive anterior impingement test on clinical examination, and radiographic evidence of cam and/or pincer morphology. Cam morphology was defined radio-graphically as an alpha angle >55°, and pincer morphology was defined as either a lateral center edge angle >39° or presence of a crossover sign. A physical examination was performed on all control participants prior to testing to confirm the absence of any hip related clinical signs or symptoms associated with FAIS (21). All participants, with the exception FAIS patients, were excluded if they answered yes to any of the following during a pre-test phone screen: (1) pain in any lower extremity joint or low back, (2) history of lower extremity or low back injury within the last 6 months, (3) any history of lower extremity fracture or surgery, (4) history of congenital hip disorders, (5) any systemic disorders that limit the participation in the study protocol.

### Hip Arthroscopic Surgery

All FAIS patients underwent arthroscopic hip surgery by a board certified orthopedic surgeon who specializes in hip arthroscopy. Hip arthroscopy was performed with the patient in the supine position. Traction was applied under fluoroscopic guidance and the anterolateral and mid-anterior portals were established. A capsulotomy was created to address injury to the central compartment such as acetabular rim trimming for pincer type impingement and acetabular labral repair. Traction was then released and the peripheral compartment was accessed to perform osteo-chondroplasty of the femoral head and neck junction in cases of cam type morphology. A dynamic examination was performed to ensure no residual impingement exists during motion. The capsule was then repaired at the completion of the case. All patients underwent a standard 4 phased postoperative rehabilitation program, which was tailored to each individual's patients progress during physical therapy (24).

### Gait Data Acquisition

Three-dimensional (3D) position data were collected at 100 Hz using a 12 camera motion capture system (Vicon, London, UK) and ground reaction force data were sampled simultaneously at 1,000 Hz using 2 floor embedded force plates (AMTI Corp., Watertown, MA). A total of 46 retroreflective markers were placed on the following anatomical locations: sternal notch, C7 spinous process, T10 spinous process, bilateral posterior superior iliac spines, bilateral anterior superior iliac spines, bilateral iliac crest, bilateral greater trochanters, bilateral medial and lateral epicondyles of the knee, bilateral medial and lateral malleoli, bilateral 5th metatarsal bases, bilateral bases of the 2nd metatarsals, bilateral base of the 1st metatarsals. Rigid plastic clusters containing 4 markers each were placed on the lateral aspects of the thighs and shanks, and clusters containing 3 markers each were affixed to the heels, of both legs. A static standing trial was collected with all markers to define the segment parameters and estimate joint centers. The medial and lateral knee epicondyle, malleoli, and greater trochanter markers were all removed prior to motion testing. All markers were places by a single examiner with 20 years of clinical experience as physical therapist. Intra-rater reliability for hip kinematics was assessed in a subsample of 5 subjects who underwent motion analysis testing on 2 occasions. with 7 days between the sessions. The average intra-class correlation coefficient (ICC_3, 3_) for peak joint kinematics was 0.75 with a standard error of the measurement of 2.15°. All participants walked along a 10-meter walkway at a self-selected walking speed. A self-selected walking speed was chosen because kinematic patterns were the variables of interest and imposing a walking speed constraint could influence a person's self-selected and preferred movement strategy.

### Gait Data Processing and Analysis

A successful gait trial was one where the foot fell completely on the force plate. Heel strike was defined by vertical ground reaction force (vGRF) exceeding 15 N and toe off was defined by vGRF <15 N after initial contact. The next consecutive heel strike was used to define a full gait cycle (i.e., stride) and was identified using a heel marker coordinate based algorithm. The algorithm identified the global minimum vertical position of the heel marker after the previous toe off (25).

Raw position and ground reaction force data were processed in Visual 3D (C-Motion, Inc, Rockville, MD). A 4th order Butterworth filter with a cutoff frequency of 6 Hz was used to filter raw position and ground reaction force data (26, 27). An 8-link segment kinematic model was built in Visual 3D using the filtered position data and by using a CODA pelvis segment (Charnwood Dynamics Ltd. Leicestershire, UK). Gait speed for each trial was defined based on the average velocity (m·s^−1^) of the center of mass (CoM) during a full stride of gait. Gait speed was defined as the average gait velocity from the 3rd to 5th successful trials collected. For a trial to be included in the analysis, the speed had to fall within 5% above or below the average gait speed. Hip joint angles were expressed according to a Cardan rotation sequence of the thigh segment relative to the pelvis segment such that the X-Y′-Z″ rotation sequence represented the medial-lateral, anterior-posterior, and superior-inferior directions. All hip joint angles during the walking trials were calculated in reference to the static standing trial. A total of five walking trials per side were collected for each subject.

### Joint Kinematic Covariation Analysis

A kinematic covariation analysis was conducted on each trial of gait for all subjects. For each walking trial, the 3D hip joint angles in each plane (*n* = 3, sagittal, frontal, and axial) were time normalized to 100 data points (m) which represents 1 stride or a full gait cycle. The data from each trial were then arranged in an n x m (3 × 100) matrix which served as the input for principal components analysis (PCA) of the 3D hip joint angle during walking. As an initial step, the z-score for each hip joint angle was calculated by subtracting the time series mean of each angle from each point and dividing by the time series standard deviation (24, 28). The rationale for z-scoring the data in this manner was to eliminate the potential bias of the waveforms magnitude in biasing the analysis toward only the spatial structure of the data. This “normalization” procedure was performed to focus more so on the temporal structure of the data used for the analysis (19, 29). Next, the covariance matrix of the waveform data was calculated, such that the covariance of each time point was determined. Next, a PCA using an eigenvector decomposition algorithm was performed on the co-variance matrix of each 3D kinematic time series (i.e., n × m matrix) (19). This PCA process was repeated for all 5 trials of the gait cycle for each subject, such that 5 PCAs were performed for each subject (i.e., 1 PCA per trial) (19). From the PCA, the eigenvectors (i.e., PC vectors) and eigenvalues (i.e., PC coefficients) were extracted from each input covariance matrix (13–16, 18, 19). The percent variance accounted for (%VAF) by each of the PCs was then determined by dividing the eigenvalue by the sum of all the eigenvalues (19). The %VAF from the first (PC1) and second (PC2) extracted PCs represent 100% of the variation in the 3D joint angle kinematic data structure (14, 15). These first 2 PCs define the orientation of the plane of kinematic covariance and therefore represent the timing or coordination among the 3D joints angles (13–16, 18, 19). In addition, to determine how much each individual plane contributed to the 3D hip joint kinematic covariation for each walking trial, the principal component scores (PC scores) were calculated (19). PC scores for each plane were calculated as the dot product between the hip joint angles at each time point of the time normalized gait cycle and the PC coefficient, which represents a projection of the original joint angle onto the plane (19). The dependent variables used for analysis were: the %VAF, which represents 3D hip joint kinematic covariation, and the PC scores for each plane, which represents the plane specific contribution to overall 3D hip joint kinematic covariation. Five-trial ensemble averages of these dependent variables were calculated for each subject and used as inputs for statistical analysis. All PCA calculations were performed using a custom written MATLAB script (Mathworks, Natick, MA).

### Patient Reported Outcomes Measures

All participants undergoing hip arthroscopic surgery completed the patient reported outcome measures (PROMs) at the time of preoperative and postoperative motion analysis testing. The hip specific PROMs of the Hip Outcome Score Activity of Daily Living Subscale and Nonarthritic Hip Score (NAHS) have both been shown to demonstrate adequate psychometric properties for measuring hip function in people with FAIS (30–33).

### Statistical Analysis

A Shapiro-Wilk test was used to determine normality and box plots were used to identify outliers for all dependent variables (i.e., %VAF and PC scores). A statistically significant outlier was defined as data exceeding 3 standard deviations from the grand mean. Independent samples *t*-tests were used to determine differences between heathy controls and FAIS patients at the preoperative time point for the dependent variables of %VAF and PC scores. Paired samples *t*-test were used to evaluate differences within FAIS patients before and 1-year after surgery for these dependent variables. All data are presented as means and standard deviations. An *a-priori* alpha level of 0.05 was set for statistical significance. All statistical testing was performed using SPSS version 22 (IBM, Chicago, IL).

## Results

Six patients undergoing hip arthroscopy were diagnosed with cam type morphology (*n* = 6) whereas 2 patients were diagnosed with mixed cam and pincer morphology (*n* = 2). All patients undergoing hip arthroscopy for FAIS demonstrated significant improvements in PROs that assessed patient reported function (Table 2). There were no between-group differences in self-selected gait speed for the preoperative FAIS group and healthy controls [Pre., 1.36 (0.10) vs. Control, 1.40 (0.16) m/s; *p* = 0.592], or within-group differences before and after arthroscopic hip surgery [Pre., 1.36 (0.10) vs. Postop 1.35 (0.06) m/s; *p* = 0.704].

**Table 2:** Patient reported outcome scores for the FAIS group at the preoperative and 1-year postoperative time points.

	**Preoperative**	**1-year postoperative**	***P***
NAHS	58.7 (14.7)	92.3 (8.2)	0.0001^‡^
HOS-ADL	0.68 (0.10)	0.91 (0.08)	0.002^†^

† *Indicates statistical significance at p < 0.005. Indicates statistical significance at p < 0.0005*.

‡ *NAHS, Non-arthritic hip score; HOS-ADL, Hip Outcome Scale Activities of Daily Living Subscale*.

### 3D Hip Joint Kinematic Covariation (%VAF)

**Figure 1:**
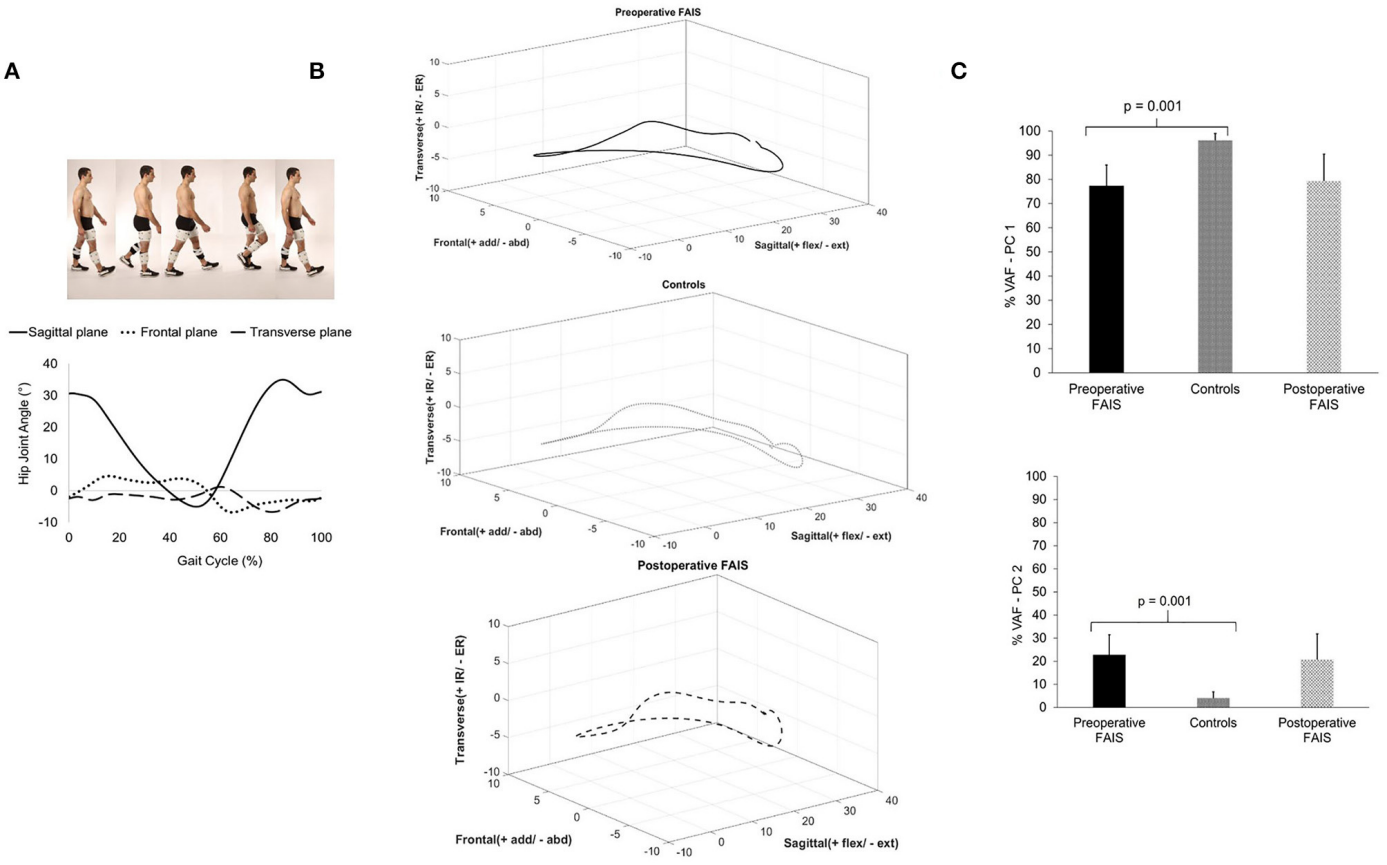
Hip joint kinematic waveforms in each plane during walking **(A)** were combined to represent the 3D hip joint angle 3D waveforms **(B)**, which were analyzed using PCA to determine the joint kinematic covariation. The kinematic control strategy of the hip joint for each group was defined as the percent variance accounted for (%VAF) **(C)**.

The trajectories of the 3D hip angles lie close to a plane (Figures 1A,B). The PC 1 %VAF was significantly less in the preoperative subjects when compared to healthy controls [FAIS, 77.2 (8.7)% vs. Control, 96.1 (2.8)%; *p* = 0.0001], whereas for PC2 the %VAF was significantly greater [22.8 (8.7) vs. 3.9 (2.8)%; *p* = 0.0001] (Figure 1C). No within FAIS group differences were found between the preoperative and postoperative time points for %VAF by PC 1 [77.2 (8.7)% vs. 79.3 (11.1)%; *p* = 0.472] or PC2 [22.7 (8.7)% vs. 20.7 (11.1)%; *p* = 0.472] (Figure 1C).

### Plane Specific Contributions (i.e., PC Scores) to 3D Hip Joint Kinematic Covariation

**Figure 2:**
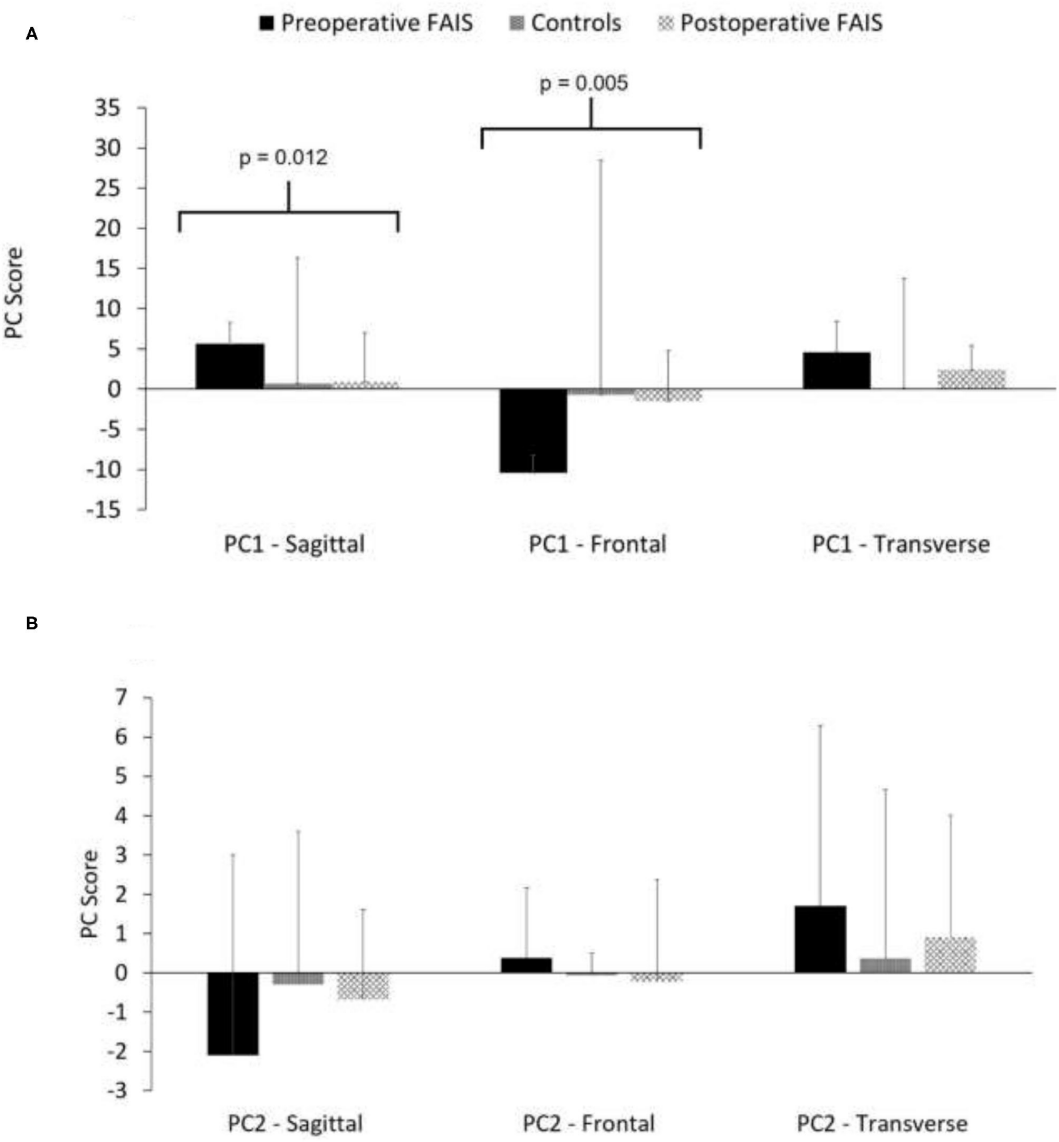
Plane specific contributions for PC1 **(A)** and PC 2 **(B)** to the hip joint kinematic covariation pattern for each group.

The PC 1 sagittal plane (Pre: 5.6 ± 2.7 vs. Post. 0.91 ± 6.1) and frontal plane (Pre: −10.4 ± 2.2 vs. Post. −1.5 ± 6.3) PC scores were different between the preoperative and postoperative time point within FAIS patients (Figure 2A). However, no differences were between the preoperative and postoperative time points within the FAIS group, and no additional differences were found for any of the PC 2 score (Figure 2B).

## Discussion

The 3D hip joint kinematic covariation strategy during gait in FAIS patients is different than healthy controls before surgery, and remains unchanged after hip arthroscopic surgery. However, after hip arthroscopic surgery for FAIS, patients did demonstrate differences the amount that the sagittal and frontal planes contributed to PC1 of the overall 3D hip joint kinematic covariation strategy, which was indicated by the difference in PC1 scores for these planes. People with FAIS demonstrate more of a planar kinematic strategy of covariation, because 2 PC's are required to explain over 90% of the variance in the kinematic covariation, whereas controls demonstrate more of a linear strategy, since only a single PC was needed to explain >90% of the variance of the kinematic covariation in the 3D hip joint angles. Additionally, the kinematic covariation strategy remains unchanged during walking 1-year after hip arthroscopic surgery. However, the contribution from the sagittal and frontal plane to PC1 were different between the preoperative and postoperative time points indicating that perhaps subtle alterations in the contribution of motion in each plane occurs after surgery. This exploratory study supports that alterations in motor control exists in FAIS patients.

Differences in the 3D hip joint kinematic covariation strategy during gait both before and 1-year after hip arthroscopic surgery indicates that motor control alterations exists in FAIS patients both before surgery and after surgery. Since joint kinematics during dynamic tasks have been shown to involve simplifying the motor control of the task by reducing redundant degrees of freedom within the musculoskeletal system (14–16, 18), the kinematic covariation framework applied to 3D joint angles waveforms provides insight to how the system controls this kinematic joint motion (19). The orientation of the plane of angular covariance, which is created by the PC1 and PC2 eigenvectors, reflects the phase relationship of the 3D joint angles thereby represents the timing of 3D coordination at the joint amongst the planes of motion (15, 18). In the current study, people with FAIS demonstrated a more planar kinematic control of 3D hip joint motion, whereas control used a linear kinematic control strategy. There are a number of reasons that these motor control strategies could have been different such as: the presence of hip pain, movement strategies adopted to avoid symptomatic impingement (34), or impairments in muscle strength or function, all of which have been found in people with FAIS. It has been shown that to change a motor pattern intense and deliberate practice is required (28, 35, 36), which may or may not be the case for gait re-training following hip arthroscopy due to the extreme variation in postoperative guidelines (37). Therefore, perhaps the reason that these motor control alterations persisting in FAIS patients after surgery, is due to the lack of specific treatments directed at modifying motor control of gait after surgery. More research is needed to investigate the underlying causes of motor control alterations in people with FAIS both before and after surgery.

The current study adds to previous studies on gait biomechanics before and after surgery for FAIS (7–11), as well as another study that investigated muscular synergy control of gait in patients with hip pathology (38). Rylander et al. reported that patients demonstrated improvement of maximal hip internal rotation and sagittal plane range motion during gait following hip arthroscopic surgery for FAIS (7). In that study, the preoperative to postoperative changes for maximum internal rotation and sagittal plane range of motion during gait were on the order of a 1.6 and 2.5 degrees, respectively (7). In the current study, the contributions to PC 1 of the 3D hip joint kinematic covariation strategy from the sagittal and frontal planes changed between the preoperative and postoperative time points in FAIS patients, however, the overall 3D hip joint kinematic covariation strategy remained unchanged. Since previous authors have shown that changes in peak joint angles between the preoperative time point and 1-year postoperative time point are small (7–9), the current findings seem consistent with these authors. Samaan et al. reported alterations in joint coordination variability between the hip and knee joints in multiple planes of motion during gait in people with acetabular chondral lesions (39). During gait these authors found that patients with acetabular cartilage lesions demonstrate a reduction in joint coordination variability between the hip and knee during loading response, but increased hip and knee coordination variability during the terminal stance and pre-swing phases of gait (39). These authors suggest that the reduced variability during loading response may indicate a strategy to facilitate stability by constraining movement, whereas greater coordination variability may indicate a compensation to avoid pain (39). Interestingly, differences in the muscle activation coordination strategy of the hip deep external rotators during the swing phase of gait were found between patients with FAIS and healthy controls (38). Diamond and colleagues showed alterations in the muscle activation timing of the deep hip external rotator muscles during the early swing phase of gait postulating that this may represent a strategy to avoid pain during the swing phase of gait (38). The findings of the current study add evidence of motor control differences between people with FAIS and healthy controls, which should be investigated further in future studies. The current findings also indicate that changes in the motor control strategy of hip joint motion after hip arthroscopic surgery for FAIS may continue to occur >1-year after surgery.

The current study is not without limitations. We acknowledge that the sample size of the study is small, however, the longitudinal design evaluating patients at 1-year after hip arthroscopy is valuable because extremely limited data exists in this area (7–9). Challenges associated with patient attrition, the time burden of marker based motion analysis testing, and limitation in number of high volume hip arthroscopy centers capable of motion analysis testing contributes to the generally small sample sizes and limited number of postoperative biomechanical studies in FAIS and (7–11). Other studies have attempted to overcome this by including patients undergoing any type of surgery for FAIS (open vs. arthroscopic), (9) and have reported on short term outcomes (10, 11). Although not ideal, the statistical analysis used in the current study is appropriate for small sample size (40). We did not use radiographic confirmation that control participants were free of cam or pincer type morphology. However, our clinical examination of healthy controls revealed that no participants demonstrated hip symptoms or clinical signs of FAIS, which would render them free of the diagnosis of FAIS, regardless as to whether they demonstrated radiographic evidence of cam or pincer morphology. Finally, we did not control for the postoperative time frame to testing, however, all FAIS patients completed formal rehabilitation and were tested within the first 18 months following hip arthroscopy.

## Conclusion

People with FAIS demonstrate alterations in the 3D hip joint kinematic control strategy during walking when compared to healthy controls, and these alterations persist within these patients after surgery. Although studies have advocated the use of movement and gait retraining in the treatment of people with FAIS before and after surgery, very little evidence exists on the impact of FAIS and treatment on motor control. This study provides preliminary evidence on kinematic control alterations in people with FAIS, which indicate that motor control impairments do exist in these patients, and treatment paradigms should consider these alterations.

## Data Availability Statement

Data will be made available upon reasonable request.

## Ethics Statement

The studies involving human participants were reviewed and approved by Marquette University Institutional Review Board. Written informed consent to participate in this study was provided by the participants' legal guardian/next of kin.

## Author Contributions

All authors listed have made a substantial, direct and intellectual contribution to the work, and approved it for publication.

## Conflict of Interest

The authors declare that the research was conducted in the absence of any commercial or financial relationships that could be construed as a potential conflict of interest.

## Publisher's Note

All claims expressed in this article are solely those of the authors and do not necessarily represent those of their affiliated organizations, or those of the publisher, the editors and the reviewers. Any product that may be evaluated in this article, or claim that may be made by its manufacturer, is not guaranteed or endorsed by the publisher.
